# Derivation of a Floquet Formalism within a Natural Framework

**DOI:** 10.1007/s10441-012-9162-4

**Published:** 2012-06-29

**Authors:** G. J. Boender, A. A. de Koeijer, E. A. J. Fischer

**Affiliations:** Central Veterinary Institute, Part of Wageningen UR, Lelystad, The Netherlands

**Keywords:** Population dynamics, Periodicity, Seasonality, Circadian cycle, Floquet theory, Floquet ratio

## Abstract

Many biological systems experience a periodic environment. Floquet theory is a mathematical tool to deal with such time periodic systems. It is not often applied in biology, because linkage between the mathematics and the biology is not available. To create this linkage, we derive the Floquet theory for natural systems. We construct a framework, where the rotation of the Earth is causing the periodicity. Within this framework the angular momentum operator is introduced to describe the Earth’s rotation. The Fourier operators and the Fourier states are defined to link the rotation to the biological system. Using these operators, the biological system can be transformed into a rotating frame in which the environment becomes static. In this rotating frame the Floquet solution can be derived. Two examples demonstrate how to apply this natural framework.

## Introduction

Many biological systems are influenced by an environment, which is periodic in nature, e.g. due to the amount of light in the circadian cycle, or the seasonal weather conditions. To describe the evolution of such biological systems the periodic forcing of the environment should be taken into account. From a mathematical perspective, the Floquet formalism provides tools to deal with such recurring patterns (Farkas 1994).

The Floquet theory deals with systems of linear differential equations with periodic coefficients, and can be used to determine the stability of equilibria (Floquet 1883). Application to the field of epidemiology has been proposed previously (Heesterbeek and Roberts 1995a, b), but the use of the Floquet theory in ecology and epidemiology remains limited today. If applied, it makes use of numerical solutions of the linearized system of periodic ODE’s (Klausmeier 2008). This is a straightforward and easily implemented method, but does not provide analytical solutions for the evolution of the biological system. For this reason the gap between the description of the evolution of the biological systems and the Floquet theory remains.

To bridge this gap, we wish to translate the Floquet formalism into a natural framework: the periodicity is dictated by the nature of the environment and is an integral part of the description of the biological system. Thus, we design a natural framework for the description of periodic causes. Furthermore, we derive the Floquet solutions of the linearized system of periodic describing the biological system within this framework and define the Floquet ratio *R*_*T*_ with the same threshold property as the basic reproduction number *R*_0_. We provide a recipe and apply it to two examples, showing that this algorithm is relatively simple and can easily be used in calculations to determine stability of a specific system.

## Conceptual Description

In this section we describe the concepts of the natural framework and the recipe for application. The full analytical description is presented in the Sect. 3, but for anyone who is only interested in practical application, it should be sufficient to read this section and continue reading in the Sect. 4.

### Key Elements of the Natural Framework for the Effect of Periodic Fluctuations

We are interested in the stability of the null equilibrium of the population, which is stable if the long term growth rate of the population is negative. Due to the rotation of the Earth, the environment of the population is forced to be periodic in time and subsequently the population growth is time dependent. The time dependency of the change in the population growth is

1 $$ \frac{\partial }{\partial t} v(\varphi _{0},t)={\bf K}(\varphi _{0},t)v(\varphi _{0},t), $$

in which $v(\varphi _{0},t)$ is the population state, **K** the periodic growth operator reflecting the influence of the environment on the population and *t* the time since start of invasion, $\varphi _{0}$ the initial phase, which depends on the observation moment in the cycle. Because in the context of this paper the Earth rotation forces the environment of the biological to be periodic in time, $\varphi _{0}$ should be interpreted as a parameter value for a variable $\varphi$ describing the rotation. In our description we undo this premature substitution and make the rotation explicit. Consequently, the environment is described by the growth operator ${\bf K}(\varphi,t).$ The periodicity of this operator is forced by the rotation of the Earth with the frequency ω_*r*_ = 2π/*T*_*r*_, in which *T*_*r*_ is the known length of the period. The different frequency components of the environment could be distinguished by means of the Fourier expansion, and therefore we can expand this operator in a Fourier series (Oppenheim et al. 1983)

2 $$ {\bf K}(\varphi, t) =\sum _{l=-\infty }^{\infty } {\bf K}_{l} {\bf F}_{l}\exp (il\omega _{ r} t), $$

in which **K**_*l*_ are the Fourier components of **K** and the Fourier operators **F**_*l*_ are defined by (Boender et al. 1998)

3 $$ {\bf F}_{l} =\exp \left(i l\varphi \right). $$

These Fourier operators are used to describe the modulation of the environment by the Earth rotation. We describe the rotation of the Earth in physical terms by an angular momentum operator **L**_*z*_, which is an infinitesimal rotation in the plane perpendicular to the axis of rotation (*z*-axis) (Alonso and Finn 1968).

4 $$ {\bf L}_{z} =-i\frac{\partial }{\partial \varphi }, $$

The periodicity of the growth operator can be removed by means of a transformation to the rotating frame, rotating with the frequency ω_*r*_ (Boender et al. 1998). The Floquet growth operator, i.e. the static growth operator in the rotating frame, is

5 $$ {\bf K}_{F} = \sum _{l=-\infty }^{\infty } {\bf K}_{l} {\bf F}_{l}-i\omega _{ r} {\bf L}_{z}. $$

The Floquet growth operator is time independent, with a correction for the rotation −*i* ω_*r*_ **L**_*z*_. The analytical derivation of this equation will be performed in the Sect. 3. The dominant eigenvalue λ_max_ of **K**_*F*_ determines the growth. If the real part *r*_max_ of dominant eigenvalue λ_max_ is larger than 0, long term growth of the population will occur. When the real part *r*_max_ is smaller than 0, the size of the population will fluctuate but still fades away to extinction. This threshold is equivalent to a description using the dominant Floquet multiplier $\hbox{ exp}\left( r_{\max} \right)$ (Heesterbeek and Roberts 1995a, b). For $\hbox{ exp} \left( r_{\max} \right) < 1$ the population will go extinct, but for $\hbox{ exp} \left( r_{\max} \right) > 1$ the population will grow. If the interval between the generations (*T*_*c*_) is exact and time independent, it is possible to construct a reproduction ratio $R = \hbox{ exp} \left( r_ {\max} T_{c}\right)$ (Wallinga and Lipsitch 2007). This well-known threshold quantity separates population growth (*R* > 1) and decline (*R* < 1). However, the generation interval *T*_*c*_ is often periodic as well, due to for instance temperature dependent mortality rates. Therefore we propose here a general threshold quantity, which is straightforward to determine: the Floquet ratio

6 $$ R_{T} = \hbox{ exp} \left( r_ {\max} T_{r}\right), $$

which is the average number of offspring of a single individual after one period. This threshold quantity separates population growth (*R*_*T*_ > 1) and decline (*R*_*T*_ < 1) in the same way as *R*.

### A Recipe for the Calculation of the Floquet Ratio

To calculate the eigenvalues of **K**_*F*_, we wish to construct a Floquet matrix of the Floquet growth operator. To this end, we have to formulate the matrices of **L**_*z*_ and **F**_*l*_. **L**_*z*_ measures the Fourier number *n* of the function $\hbox{ exp} \left(in\varphi \right)$ and therefore the matrix can be formulated as

7 $$ \left(\begin{array}{ccccccccc} \cdots&\cdots& \cdots& \cdots &\cdots & \cdots &\cdots \\ \cdots& 2 & 0 & 0 & 0 & 0 &\cdots \\ \cdots& 0 & 1 & 0 & 0 & 0 & \cdots \\ \cdots& 0 & 0 & 0 & 0 & 0 & \cdots \\ \cdots& 0 & 0 & 0 & -1 & 0 & \cdots \\ \cdots& 0 & 0 & 0 & 0 & -2 & \cdots \\ \cdots&\cdots& \cdots& \cdots& \cdots &\cdots & \cdots\\ \end{array}\right). $$

**F**_*l*_ increases the Fourier number *n* of the function $\hbox{ exp} \left(in\varphi \right)$ to *n* + *l*. Therefore the matrix for **F**_1_ can be formulated as

8 $$ \left(\begin{array}{ccccccccc} \cdots&\cdots& \cdots& \cdots &\cdots & \cdots &\cdots \\ \cdots& 0 & 1 & 0 & 0 & 0 &\cdots \\ \cdots& 0 & 0 & 1 & 0 & 0 & \cdots \\ \cdots& 0 & 0 & 0 & 1 & 0 & \cdots \\ \cdots& 0 & 0 & 0 & 0 & 1 & \cdots \\ \cdots& 0 & 0 & 0 & 0 & 0 &\cdots \\ \cdots&\cdots& \cdots& \cdots& \cdots &\cdots & \cdots\\ \end{array}\right), $$

and the matrix for **F**_*l*_ can be found by multiplication of this matrix *l* times, because **F**_*l*_ = (**F**_1_)^*l*^. According to Eqs. 5, 7 and 8 the Floquet matrix can be written as

9 $$ \left(\begin{array}{ccccccccc} \cdots&\cdots& \cdots& \cdots &\cdots & \cdots &\cdots \\ \cdots& {\bf K}_0- 2 i \omega_r {\bf I} & {\bf K}_1 & {\bf K}_2 & {\bf K}_{3} & {\bf K}_{4} & \cdots \\ \cdots& {\bf K}_{-1} & {\bf K}_0- i \omega_r {\bf I} & {\bf K}_1 & {\bf K}_2 & {\bf K}_{3} & \cdots \\ \cdots& {\bf K}_{-2} & {\bf K}_{-1} & {\bf K}_0 & {\bf K}_1 & {\bf K}_2 & \cdots \\ \cdots& {\bf K}_{-3} & {\bf K}_{-2} & {\bf K}_{-1} & {\bf K}_0 + i \omega_r {\bf I} & {\bf K}_1 & \cdots \\ \cdots& {\bf K}_{-4} & {\bf K}_{-3} & {\bf K}_{-2} & {\bf K}_{-1} & {\bf K}_0+ i 2 \omega_r {\bf I} & \cdots \\ \cdots&\cdots& \cdots& \cdots& \cdots &\cdots & \cdots \\ \end{array}\right), $$

in which the identity matrix **I** describes the rotational term *i* ω_*r*_ **L**_*z*_. In Box 1 a recipe is presented how to apply this description. In the Sect. 4 we will describe two examples.

**Box 1:** Recipe to determine the Floquet ratio

(1) Establish a time dependent growth matrix ${\bf K}(\varphi _{0} ,t)$ (see Eq. 1). This matrix describes the growth of the system (e.g. the increase of population size or the transmission and recovery causing a change in the number of infected hosts)
(2) Determine the Fourier components **K**_−*n*_ to **K**_*n*_ of the growth matrix **K** (see Eq. 2)
(3) Formulate the Floquet matrix (see Eq. 9)
(4) Calculate the eigenvalues of the Floquet matrix, if possible analytically or otherwise using a numerical approximation
(5) Compose the Floquet ratio *R*_*T*_ from the real part of the largest eigenvalue

In the Sect. 3 we will present a comprehensive derivation of the theory. Readers who wish to focus on the application can continue reading in Sect. 4.

## Theoretical Derivation

### The Natural Framework

The variable $\varphi$, as introduced in previous section, is essential for the constitution of the natural framework, because this is the variable for the description of the Earth’s rotation. For an arbitrary function $f(\varphi)$ holds $f\left(\varphi \right)=f\left(\varphi +2\pi k\right)$ with *k* an integer. This function could be expanded in terms of the Fourier states similar to a Fourier expansion (Oppenheim et al. 1983).

10 $$ \begin{aligned} f(\varphi ) &= \sum _{l=-\infty }^{\infty }f_{l} \varvec{\Upphi} _{l} (\varphi ) \\ f_{l} &=\int\limits_{-\pi }^{\pi }f(\varphi )\varvec{\Upphi} _{l}^{*} (\varphi )d\varphi, \\ \end{aligned} $$

with as base the Fourier states $\varvec{\Upphi}_{l}={\tfrac{1}{\sqrt{2\pi } }} \hbox{ exp} \left(il\varphi \right)$ (Alonso and Finn 1968; Boender et al. 1998). The Fourier states have the property $\varvec{\Upphi} _{l}^{*} \left(\varphi \right)={\tfrac{1}{\sqrt{2\pi } }} \hbox{ exp} \left(-il\varphi \right)=\varvec{\Upphi} _{-l} \left(\varphi \right)$. From Eq. 10 it follows that the Fourier states are normalized, because for the special case $f\left(\varphi \right)=\varvec{\Upphi} _{n} \left(\varphi \right)$, the coefficients *f*_*l*_ equal the Delta function δ_*nl*_, which is 1 for n = *l* and 0 otherwise. Therefore, $\varvec{\Upphi} _{n} \left(\varphi \right)$ constitute an orthonormal base for the description of functions and operators. The unit impulse or Dirac Delta is one the functions we applied further on (Oppenheim et al. 1983). This function is defined by its transformation property of an arbitrary function *g*

11 $$ \int\limits _{-\pi }^{\pi }g\left(\varphi \right)\delta (\varphi -\varphi _{0} )d\varphi =g\left(\varphi _{0} \right). $$

By means of a expansion, this Dirac Delta function can be expressed in terms of the Fourier states (Boender et al. 1998)

12 $$ \begin{aligned} \delta (\varphi -\varphi _{0} )&=\sum _{m=-\infty }^{\infty }\left(\int\limits _{-\pi }^{\pi }\delta (\varphi -\varphi _{0} )\varvec{\Upphi} _{m}^{*} (\varphi )d\varphi \right)\varvec{\Upphi} _{m} \left(\varphi \right)\\ &=\sum _{m=-\infty }^{\infty }{\tfrac{1}{\sqrt{2\pi } }} \hbox{ exp} (-im\varphi _{0} )\varvec{\Upphi} _{m} \left(\varphi \right),\\ \end{aligned} $$

according to Eq. 10. Furthermore, the Angular momentum operator **L**_*z*_ and the Fourier operator **F**_*p*_ can be formulated with respect to the Fourier states. The eigenvalue equation for the angular momentum operator becomes (Alonso and Finn 1968; Boender et al. 1998)

13 $$ {\bf L}_{z} \varvec{\Upphi} _{l} (\varphi )=l\varvec{\Upphi} _{l} (\varphi ). $$

Therefore, $\varvec{\Upphi}_{l} (\varphi )$ are the eigenstates of **L**_*z*_. When **F**_*p*_ are applied to $\varvec{\Upphi}_{l}(\varphi)$, this leads to

14 $$ \begin{aligned} {{\bf F}_{p} \varvec{\Upphi} _{l} (\varphi )} &= {{\tfrac{1}{\sqrt{2\pi } }} \hbox{ exp} \left(ip\varphi \right)\hbox{ exp} \left(il\varphi \right)} \\ &= {\varvec{\Upphi} _{l+p} (\varphi ).} \\ \end{aligned} $$

This operator causes an increase in the order of the function, i.e. *l* is transformed into *l* + *p*. We will use this property in the derivations to calculate the multiplication of Fourier components.

To finish the picture, we derive the relation of **L**_*z*_ and **F**_*p*_, using the expansion of the exponential function. This leads to the following relation for the Fourier operator

15 $$ \begin{aligned} &{\hbox{exp}\left(i\omega _{ r} {\bf L}_{z} t\right) {\bf F}_{p} \hbox{exp}\left(-i\omega _{ r} {\bf L}_{z} t\right)\xi } \\ &={\left(\sum _{j=0}^{\infty }\frac{\left(i\omega _{ r} t\right)^{n} }{n!} \left({\bf L}_{z} \right)^{n} \right){\bf F}_{p} \hbox{ exp} \left(-i\omega _{ r} {\bf L}_{z} t\right)\xi } \\ &={{\bf F}_{p} \left(\sum _{j=0}^{\infty }\frac{\left(i\omega _{ r} t\right)^{n} }{n!} \left(p+{\bf L}_{z} \right)^{n} \right)\hbox{ exp} \left(-i\omega _{ r} {\bf L}_{z} t\right)\xi } \\ &={{{\bf F}_{p} p} \hbox{ exp} \left(ip \omega _{ r} t\right) \hbox{exp} \left(i \omega _{ r} {\bf L}_{z} t\right)\hbox{ exp} \left(-i\omega _{ r} {\bf L}_{z} t\right)\xi } \\ &={{\bf F}_{p} \hbox{ exp} \left(ip \omega_{ r} t\right)\xi }, \\ \end{aligned} $$

in which ξ is an arbitrary state. In this derivation we use the expansion of an exponential function, the property $\hbox{ exp} \left(i\omega _{ r} {\bf L}_{z} t\right)\hbox{ exp} \left(-i\omega _{ r} {\bf L}_{z} t\right)=1$ and the following relation

16 $$ \begin{aligned} {{\bf L}_{z} {\bf F}_{p} \xi } &= {\left(-i\frac{\partial }{\partial \varphi } \right)\hbox{ exp} \left(ip\varphi \right)\xi } \\ & = {p\hbox{ exp} \left(ip\varphi \right)\xi +\hbox{ exp} \left(ip\varphi \right)\left(-i\frac{\partial }{\partial \varphi } \right)\xi} \\ &= {{\bf F}_{p} \left(p + {\bf L}_{z} \right)\xi. }\\ \end{aligned} $$

This natural framework facilitates the description of the population dynamics. The population could be expressed in terms of the Fourier states $\varvec{\Upphi}_{l} \left(\varphi \right)$. This population is modulated by the environment, which is expressed in terms of the Fourier operators **F**_*p*_. The environment is forced to be periodic by the rotation of the Earth, which is described by the angular momentum operator **L**_*z*_.

### Derivation of the Floquet Solution within the Natural Framework

We apply this natural framework to determine the growth of a population in a time periodic environment. The Floquet formalism unravels the solutions into two components: the long term behavior of the system and separated this from the short term (periodic) behavior of the system (Floquet 1883). The method is straight forward, in the sense that the long term behavior of a system is described directly by the Floquet exponents (Farkas 1994). If the largest of the Floquet exponents is larger than 0 the population will grow.

According to Eq. 1, The biological system is described by a population state $v(\varphi _{0},t)$ and the effect of the environment on this biological system by a growth operator ${\bf K}(\varphi_{0},t)$, in which the initial phase $\varphi _{0}$ is a constant. We transform the non-differentiable constant $\varphi_{0}$ into a differentiable variable $\varphi$ to enable a description of the effect of the rotation on the biological system. This phase variable $\varphi$ can be transformed back to the initial phase $\varphi _{0}$, accordingly

17 $$ \begin{aligned} v(\varphi _{0},t) &= \int \limits_{-\pi }^{\pi }v(\varphi,t)\delta (\varphi -\varphi _{0} )d\varphi \\ & = \sum^{\infty}_{m= -\infty}{\frac{1}{\sqrt{2 \pi}} \hbox{ exp}{\left(- i m \varphi_{0} \right) \int\limits^{\pi}_{-\pi}{v(\varphi,t) \varvec{\Upphi}_{m}\left(\varphi\right)d\varphi}}} \\ {\bf K} (\varphi _{0},t) v(\varphi _{0},t) & = \sum^{\infty}_{m= -\infty}{\frac{1}{\sqrt{2 \pi}} \hbox{ exp}{\left(- i m \varphi_{0} \right) \int\limits^{\pi}_{-\pi}{{\bf K} (\varphi,t) v(\varphi,t) \varvec{\Upphi}_{m}\left(\varphi\right)d\varphi}}}, \\ \end{aligned} $$

in which Eqs. 11 and 12 are applied. The mathematical solution of Eq. 1 is presented by Floquet (1883). This solution is applied in physics to solve the Schrödinger equation for a period Hamiltonian operator, which describes the energy of a system leading to the Floquet Theory (Boender et al. 1998; Shirley 1965). We will now apply this theory to the field of population biology. We present the algebraic derivation of disentanglement of periodic changes and the long term growth.

At the start of the invasion one typical individual is present. This initial state will be denoted by *v*_0_. An invasion can start at any time during the season. Therefore, this state *v*_0_ is independent of *t* and $\varphi_{0}$. According to Eq. 17, Eq. 1 can be transformed into a equation of which $v(\varphi,t)$ is the solution

18 $$ \begin{aligned} \frac{\partial }{\partial t} v(\varphi,t)&={\bf K}(\varphi,t)v(\varphi,t) \\ \frac{\partial }{\partial t} {{\bf U}}(\varphi,t)v_{0}& ={\bf K}(\varphi,t){{\bf U}}(\varphi,t)v_{0},\\ \end{aligned} $$

in which the evolution operator ${\bf U}(\varphi,t)$ describes the development of the biological system in time, and is defined by

19 $$ v(\varphi,t)={{\bf U}}(\varphi,t)v_{0}. $$

In the following we elaborate on the operator part of Eq. 18, resulting in disentanglement of the growth operator ${\bf K}(\varphi,0)$ and the rotating period ω_*r*_. Substitution of the outcome in Eq. 18 transforms the whole equation into a rotation frame, in which the growth operator becomes time independent. As a consequence this equation can be solved. After substitution of this solution in Eq. 17, the variable $\varphi$ is transformed back into a constant $\varphi_{0}$, leading to an explicite expression for the population state $v(\varphi _{0},t)$.

We use the Eqs. 15 and 2 to transform Eq. 18 into a constant growth operator in a rotating frame,

20 $$ \begin{aligned} &\frac{\partial }{\partial t} {\bf U}(\varphi,t)-{\bf K}(\varphi ,t){\bf U}(\varphi,t) = \\ &{\frac{\partial}{\partial t}} {\bf U}(\varphi,t)-\left({\sum_{p=-\infty}^{\infty}} {\bf K}_{p} \hbox{ exp}(ip\varphi +ip\omega_{r} t) \right){\bf U}(\varphi,t)= \\ &\frac{\partial }{\partial t} {\bf U}(\varphi,t)-\left(\hbox{ exp} (i\omega _{r} {\bf L}_{z} t)\left(\sum _{p=-\infty}^{\infty}{\bf K}_{p} {\bf F}_{p} \right)\hbox{ exp} (-i\omega _{r} {\bf L}_{z} t)\right){\bf U}(\varphi,t)=\\ &\frac{\partial}{\partial t} {\bf U}(\varphi ,t)-\left(\hbox{exp}(i\omega_{r}{\bf L}_{z} t){\bf K}(\varphi ,0)\hbox{ exp} (-i\omega_{r} {\bf L}_{z} t\right){\bf U}(\varphi ,t).\\ \end{aligned} $$

Thus, the growth operator ${\bf K}(\varphi,0)$ is disentangled from the rotation with ω_*r*_ around the axis of rotation (*z*-axis), which is described by the exponential operator $\hbox{ exp} \left(i\omega_{r} {\bf L}_{z} t\right)$. To transform the total equation into the rotating frame we substitute the Floquet evolution operator ${\bf U}_{ F}(\varphi,t)=\hbox{ exp} \left(-i\omega_{ r} {\bf L}_{z} t\right){\bf U} (\varphi,t)$ into Eq. 20, leading to

$$ \begin{aligned} &{\frac{\partial }{\partial t} \hbox{ exp} (i\omega_{ r} {\bf L}_{z} t){{\bf U}}_{ F} (\varphi,t)-(\hbox{ exp} (i\omega_{ r} {\bf L}_{z} t){\bf K}(\varphi,0){{\bf U}}_{ F} (\varphi,t)=} \\ &{i\omega_{r} {\bf L}_{z} \hbox{ exp} (i\omega_{ r} {\bf L}_{z} t){{\bf U}}_{ F} (\varphi,t)+\hbox{ exp} (i\omega _{ r} {\bf L}_{z} t)\left(\frac{\partial }{\partial t} {{\bf U}}_{ F} (\varphi ,t)-{\bf K}(\varphi,0){{\bf U}}_{ F} (\varphi,t)\right)=} \\ &{\hbox{ exp} (i\omega _{ r} {\bf L}_{z} t)\left(\frac{\partial }{\partial t} {{\bf U}}_{ F} (\varphi,t)-({\bf K}(\varphi ,0)-i\omega _{ r} {\bf L}_{z} ){{\bf U}}_{ F} (\varphi ,t)\right)=} \\ &{\hbox{ exp} (i\omega _{ r} {\bf L}_{z} t)\left(\frac{\partial }{\partial t} {{\bf U}}_{ F} (\varphi,t)-{\bf K}_{ F} (\varphi ){{\bf U}}_{ F} (\varphi,t)\right)}.\\ \end{aligned} $$

Because these expressions equal zero according to Eq. 18, this leads to a directly solvable equation

21 $$ \frac{\partial }{\partial t} {{\bf U}}_{ F} (\varphi,t)={\bf K}_{ F} (\varphi ){{\bf U}}_{ F} (\varphi,t), $$

in which **K**_*F*_ is the Floquet growth operator, according to Eq. 5. Equation 21 provides a solution for the Floquet evolution operator, ${\bf U}_{ F} (\varphi,t)=\hbox{ exp} \left({\bf K}_{ F} (\varphi )t\right)$. Using the transformed Eq. 21, the solution of Eq. 18 is given by

22 $$ \begin{aligned} v(\varphi,t) = {{\bf U}}(\varphi,t)v_{0} \\ &=\hbox{ exp} \left(i\omega _{ r} {\bf L}_{z} t\right){{\bf U}}_{ F} (\varphi,t)v_{0} =\hbox{ exp} \left(i\omega _{ r} {\bf L}_{z} t\right)\hbox{ exp} \left({\bf K}_{ F} (\varphi )t\right)v_{0}.\\ \end{aligned} $$

This is the operator presentation of the Floquet solution within the natural framework. Periodic changes and the long term growth are disentangled in this solution, in which *i*ω_*r*_ **L**_*z*_ describes the periodic changes and ${\bf K}_{F}(\varphi)$ the long term growth.

### The Threshold Quantity for Long Term Growth

Because the Floquet growth operator, **K**_*F*_, contains only the Angular momentum operator **L**_*z*_ and the Fourier operator **F**_*p*_ (see Eq. 22), the eigenvalues λ_*j*_ and eigenstates $\psi_{j}(\varphi )$ of **K**_*F*_ can be calculated. To do so, the initial state should be expressed in terms of these eigenstates:

23 $$ v_{0} =\sum _{j}v_{0j} \psi _{j} (\varphi ). $$

In Eq. 10 it is shown that the eigenstates $\psi _{i}(\varphi)$ can be expanded in terms of Fourier states, $f_{il}\varvec{\Upphi}_{l}(\varphi)$,

24 $$ v_{0} =\sum _{j}\sum _{l=-\infty }^{\infty }v_{0j} f_{jl} \varvec{\Upphi} _{l} (\varphi ), $$

where *f*_*jl*_ is the *l*th Fourier coefficient of the Fourier expansion for the *j*th eigenstate. Substitution of Eq. 24 in Eq. 17 gives

25 $$ \begin{aligned} &v(\varphi _{0},t) \\ &=\sum _{m=-\infty}^{\infty }{\tfrac{1}{\sqrt{2\pi }}} {e}^{-im\varphi _{0}}\int\limits_{-\pi }^{\pi }\left({e} ^{i\omega _{r}{\bf L}_{z} t}\left(\sum _{j}{e}^{{\bf K}_{ F} (\varphi )t}v_{0j} \psi _{j} (\varphi ) \right)\right)\varvec{\Upphi} _{m} \left(\varphi \right)d\varphi\\ &=\sum _{n=-\infty }^{\infty }{\tfrac{1}{\sqrt{2\pi } }} {e}^{in\varphi _{0}}\int\limits_{-\pi }^{\pi }\left(\sum _{j}{e}^{\lambda _{j} t}\sum _{l=-\infty }^{\infty }{e}^{i\omega _{ r} l t}v_{0j} f_{jl} \varvec{\Upphi} _{l} (\varphi ) \right)\varvec{\Upphi} _{-n} \left(\varphi \right)d\varphi\\ &={\tfrac{1}{\sqrt{2\pi } }} \sum _{n=-\infty }^{\infty }\sum _{j}v_{0j} f_{jn} {e}^{in(\varphi _{0} +\omega _{ r} t)}{e}^{\lambda _{j} t}, \end{aligned} $$

which is analogue to the Shirley formula (Shirley 1965). In the derivation the eigenvalue equation of the angular momentum operator in Eq. 13 and the orthonormality of the Fourier states according to Eq. 10 are applied. Equation 1 is now given in terms of Fourier coefficients, *f*_*jl*_, and Floquet exponents, λ_*j*_. If the real part *r*_max_ of dominant eigenvalue λ_max_ is larger than 0, long term growth of the population will occur. When the real part *r*_max_ is smaller than 0, the size of the population will fluctuate but still fades away to extinction.

### Approximation Methods for the Calculation of the Growth Rate

To calculate the eigenvalues of the Floquet operator, we applied approximation methods for the eigenvalues of this operator, which are are regularly applied in physics (Leskes et al. 2010). In this section we follow this approach and apply this to biological systems. To determine the eigenvalues, we have to find the diagonalized Floquet operator **D**_*F*_, which has the following property

26 $$ \varvec{\Uplambda}_{ F}={{\bf D}}_{ F}^{-1} {\bf K}_{ F} {{\bf D}}_{ F}=\varvec{\Uplambda}_{ 0}{\bf F}_{ 0}-i\omega _{ r} {\bf L}_{z}, $$

in which **D**_*F*_ the diagonalization Floquet operator $\varvec{\Uplambda}_{ F}$ is the diagonalized Floquet growth operator, and $\varvec{\Uplambda}_{0}$ it’s zero Fourier component. The diagonalization Floquet operator can be defined by

27 $$ {{\bf D}}_{F}=\hbox{ exp} \left({{\bf S}}_{ F}\right). $$

This can be used to expand Eq. 26 according to

28 $$ {{\bf D}}_{ F}^{-1} {\bf K}_{ F} {{\bf D}}_{ F}={\bf K}_{ F}+[{\bf K}_{ F},{{\bf S}}_{ F}]+\frac{1}{2}[[{\bf K}_{ F},{{\bf S}}_{ F}],{{\bf S}}_{ F}]\ldots, $$

in which the commutator of two operators **A** en **B**:  [**A**, **B**] = **A** **B** − **B** **A** is applied. From Eqs. 3 and 16 the following commutation relations can be deduced:

29 $$ \begin{aligned} {[{\bf L}_{z},{\bf F}_{p}]}&=p{\bf F}_{p}\\ [{\bf F}_{m},{\bf F}_{n}]&=0, \\ \end{aligned} $$

in which is used **F**_*m*_ **F**_*n*_ = **F**_*m*+*n*_. In the diagonalisation procedure, we expand the operator **S**_*F*_ = **S**_*F*_^(1)^ + **S**_*F*_^(2)^… into several orders. To diagonalize the Floquet growth operator in first order, the (off-diagonal) terms of Eq. 5 should be removed, leading to

30 $$ {{\bf S}}_{ F}^{(1)}= \sum _{n\neq 0}\frac{{\bf K}_{n}}{i n \omega _{ r}} {\bf F}_{ n}. $$

This removes the off-diagonal blocks of **K**_*F*_ as a result of the relation

31 $$ [-i n \omega _{ r}{\bf L}_{z},{{\bf S}}_{ F}^{(1)}]= -\sum _{n\neq 0}{\bf K}_{n} {\bf F}_{ n}, $$

according to Eq. 29. Substitution of this in Eq. 28 gives

32 $$ \begin{aligned} \varvec{\Uplambda}_{ F}&= {\bf K}_{0} {\bf F}_{0} -i\omega _{ r} {\bf L}_{z} +\sum_{n \neq 0}^{} \frac{\left[{\bf K}_{-n},{\bf K}_{n} \right]}{i 2 n \omega_r} {\bf F}_{0}+\\ &\sum_{n \neq 0} \frac{\left[{\bf K}_{0},{\bf K}_{n} \right]}{i n \omega_r} {\bf F}_{n}+ \sum_{n \neq 0,p \neq 0,p \neq n}^{} \frac{\left[{\bf K}_{p-n},{\bf K}_{n} \right]}{i 2 n \omega_r}{\bf F}_{p}+\ldots\\ \end{aligned} $$

The two latter terms are the higher order off-diagonal terms. If [**K**_*p*_,**K**_*n*_] = 0 for arbitrary *p* and *n*, these terms would disappear and $\varvec{\Uplambda}_{0}={\bf K}_{0}$. If the size of **K**_*n*_ is much smaller than the rotation frequency, i.e. $\left|{\bf K}_{n}\right| \ll n \omega_r$, then the higher order terms in Eq. 32 are negligible. If this is not the case, the diagonalization procedure should be extended to the second order leading to

33 $$ {{\bf S}}_{ F}^{(2)}=\sum_{n \neq 0}^{} \frac{\left[{\bf K}_{0} ,{\bf K}_{n} \right]}{\left(i n \omega_r\right)^{2}} {\bf F}_{n}+ \sum_{n \neq 0,p \neq 0,p \neq n}^{} \frac{\left[{\bf K}_{p-n} ,{\bf K}_{n} \right]}{2 n p \left(i \omega_r\right)^{2}}{\bf F}_{p}, $$

in which the same procedure is applied as was done in the first step in Eq. 30. Substitution of Eqs. 30 and 33 into Eq. 28 would lead to a Floquet growth operator which is diagonal up to second order. This procedure should be repeated until desired convergence is obtained. In the next section we will provide two examples where we apply this formalism for the analytical determination and the numerical determination of *R*_*T*_.

## Application

In this section we illustrate the recipe for the determination of the Floquet ratio (see Box 1) by means of two simple models. For a more complex model we refer to a study of Rift Valley fever (Fischer et al. in prep).

### An Example for Analytical Determination of the Floquet Ratio *R*_*T*_

#### Establish a Time Dependent Growth Matrix ${\bf K}(\varphi _{0},t)$

In this subsection we describe an ecological example: the establishment of an exotic species. The establishment of an exotic species requires that the equilibrium without the new species is unstable. Seasonality will have an effect on a new species. Especially seasonal temperature changes strongly affects population growth of ectothermic animals such as arthropods, fish, amphibians and reptiles. To illustrate our Floquet method, we will use the example of a species with a temperature dependent intrinsic growth rate, $g(\varphi_0, t)$ (see e.g. Delatte et al. 2009). The intrinsic growth rate is the difference between the birth rate and the mortality rate. For a model of this biological system Eq. 1 has the form,

34 $$ {\frac{\partial}{\partial t}} v(\varphi_{0},t)= {\bf K}(\varphi _{0} ,t)v(\varphi _{0},t) = g(\varphi_{0},t)v(\varphi_{0},t) $$

The average daily temperature follows a sinus function with the periodicity $\omega_{r} = \frac{2\pi}{T_{r}}$, in which *T*_*r*_ = 1 year. For this simple example, we assume that the intrinsic rate of increase is linear with temperature. The intrinsic growth rate of increase has the form $g(\varphi_{0},t)= g_{mean} + g_{amp} \sin(\omega_r t)$, where *g*_*mean*_ is the mean growth rate and *g*_*amp*_ the amplitude of the intrinsic growth rate in time.

#### Determine the Fourier Components **K**_−*n*_ to **K**_*n*_ of the Growth Matrix ${\bf K}(\varphi _{0},t)$

The expression $g(\varphi_{0},t)$ could be expanded in Fourier series $-\frac{1}{2i} g_{amp} \hbox{ exp}{(-(\omega_r t))}+g_{mean}+\frac{1}{2i} g_{amp} \hbox{ exp}{(\omega_r t)}$. As a consequence, the Fourier coefficients are $K_{-1}=-\frac{1}{2i} g_{amp},\,K_{0}= g_{mean}$, and $K_{1}=\frac{1}{2i} g_{amp}$, according to Eq. 2.

#### Formulate the Floquet Matrix (see Eq. 9)

In this case the matrix **K**_*n*_ equals the number *K*_*n*_ and the identity matrix **I** equals 1.

#### Calculate the Eigenvalues of the Floquet Matrix

Because *K*_−1_, *K*_0_, and *K*_1_ are coefficients in this example, they all commute, i.e. [*K*_*p*_,* K*_*n*_] = *K*_*p*_ *K*_*n*_ − *K*_*n*_ *K*_*p*_ = 0. According to Eq. 20, the eigenvalues of the diagonalized matrix is

35 $$ \lambda = g_{mean}-i n {\omega _r}. $$

#### Compose the Floquet Ratio *R*_*T*_ from the Real Part of the Largest Eigenvalue

*r*_max_ = *g*_*mean*_ is the real part of the dominant eigenvalue. Therefore the threshold quantity could be constructed, according to 6 and the Floquet ratio becomes

36 $$ R_{T} = \hbox{ exp} \left(g_{mean} T_{r} \right). $$

If *R*_*T*_ > 1 i, the null equilibrium is unstable. Furthermore, the population cannot grow if *R*_*T*_ < 1. Obviously for this simple example, the Floquet ratio is larger than one if the growth rate is positive.

### An Example for Numerical Determination of the Floquet Ratio *R*_*T*_

#### Establish a Time Dependent Growth Matrix ${\bf K}(\varphi _{0},t)$

We illustrate this by means of a epidemiological example: a vector-borne infection. In the field of epidemiology, the class of vector-borne infectious diseases are most profoundly forced by seasonal changes in the rates. Here we discuss a simple periodic model of a vector-borne infection describing one vector population and one host population, in which all parameters are time independent with exception of the transmission rate, β(*t*), and the mortality rate of the vector μ_*x*_(*t*). For such a model, Eq. 1 has the form,

37 $$ \begin{aligned} &\frac{\partial }{\partial t} v(\varphi _{0},t)={\bf K}(\varphi _{0},t)v(\varphi _{0},t)\\ &{\frac{\partial}{\partial t}\left(\begin{array}{l} x(\varphi_{0},t) \\ y(\varphi_{0},t) \end{array}\right)=\left(\begin{array}{ll} -\mu_{x}(t) & {\frac{\beta(t)}{N_{h}}} \\ \beta(t) & -\mu_{y} \end{array}\right)\left(\begin{array}{l} x(\varphi_{0},t) \\ y(\varphi_{0},t) \end{array}\right)}\\ \end{aligned} $$

in which *x* and *y* are the number of infected vectors and hosts, and μ_*x*_(*t*) is vector mortality and μ_*y*_ is host mortality. In this example we investigate a seasonal effect with the periodicity $\omega_{r} = \frac{2\pi}{T_{r}}$, in which *T*_*r*_ = 1 year. The transmission rate is a function of the seasons by average daily temperature (24 h) due to maturation of the eggs, the size of the population peaks twice during the period, and each day has a fluctuation in vector activity.

38 $$ \beta(t) = \beta_{mean }+\beta_{temp} \sin \left(\omega _r t\right) +\beta_{pop} \sin \left(2\omega _r t\right)+\beta_{day} \sin \left(365\omega _r t\right), $$

in which β_*mean*_ is the mean transmission rate and the amplitudes of the transmission rate are due to the temperature β_*temp*_ and due to the size of the population β_*pop*_, and the daily amplitude of the vector activity is β_*day*_. The mortality rate is only a simple function of temperature with a mean μ_*mean*_ and amplitude μ_*temp*_:

39 $$ \mu_{x}\left(t\right)= \mu_{mean} + \mu_{temp}\sin \left({\omega _r}t\right), $$

#### Determine the Fourier Components **K**_−*n*_ to **K**_*n*_ of the Growth Matrix ${\bf K}(\varphi _{0},t)$

The function of the mortality rate can easily be expanded in a finite Fourier series using the relation $\sin \left({\omega _r}t\right)= \frac{\hbox{ exp} \left({i \omega _r}t\right)-\hbox{ exp} \left({-i\omega _r}t\right)}{2i}$ (see previous example). The Fourier components for the transmission rate are ±1,  ±2 and ±365. We neglect the daily rhythm of transmission (±365), because this will most likely have a small amplitude and is effective on a different time scale. Using Eqs. 24, 37 and 39 the different Fourier components **K**_*l*_ can be calculated:

40 $$ \begin{aligned} {\bf K}_{0} &=\quad\left(\begin{array}{ll} {-\mu_{mean}} & {\frac{\beta_{mean}}{N_{h}}}\\ {\beta_{mean}} & {-\mu_{y}} \end{array}\right)\\ {\bf K}_{1} &= {\frac{1}{2i}} \left(\begin{array}{ll} {-\mu_{temp}} & {\frac{\beta_{temp}}{N_{h}}}\\ {\beta_{temp}} & {0} \end{array}\right)\quad=-{\bf K}_{-1}\\ {\bf K}_{2} &= {\frac{1}{2i}}\left(\begin{array}{ll} {0} & {\frac{\beta_{pop}}{N_{h}}} \\ {\beta_{pop}}& {0} \end{array}\right)\qquad\,=-{\bf K}_{-2}\\ \end{aligned} $$

#### Formulate the Floquet Matrix (see Eq. 9)

The Fourier coefficients are now represented as matrices, according to Eq. 40.

#### Calculate the Eigenvalues of the Floquet Matrix

Diagonalization of this Floquet matrix will provide the eigenvalues of this matrix. In this example, the calculation of the eigenvalues needs to be approximated by numerical techniques. In principle the Floquet matrix is infinite. For numerical calculation, the matrix must be truncated to determine the eigenvalues. This truncation will introduce numerical errors. According to Eq. 26, eigenvalues are not unique numbers, but should follow the pattern λ_0_ − *i* *n* ω_*r*_ (Farkas 1994; Shirley 1965). If the truncated Floquet matrix is extended, higher orders of *n* should appear. Eigenvalues which do not follow this pattern, are not the sought values, but numerical errors caused by the truncation. The eigenvalues of the Floquet matrix are in general complex, because in the evolution of interacting subpopulations, oscillations could occur. The imaginary part of λ_0_ will reflect these oscillations. However, the real part of the dominant eigenvalue determines whether growth occurs. For this reason we focus on the real part. The maximum value of the real parts of the eigenvalue *r*_max_ determines the evolution of the epidemic.

#### Compose the Floquet Ratio *R*_*T*_ from the Real Part of the Largest Eigenvalue

The Floquet ratio is given in Eq. 36. If *R*_*T*_ > 1 i, major outbreaks could occur and if *R*_*T*_ < 1 only minor outbreaks could occur.

## Discussion

We derived a methodology to determine the stability of the null-equilibrium for biological systems which are periodically forced by the environment. This formalism can be used for simple models in which one or a few parameters are forced to change periodically in time and for complex models including many forced time-periodic parameters. For simple models analytical results for the eigenvalues of the Floquet growth matrix *K*_*F*_ can be achieved. The real part of the dominant eigenvalues *r*_max_ of *K*_*F*_ leads straightforward to the Floquet ratio *R*_*T*_, which has the well-known properties of a threshold quantity. For complex models a numerical calculation of *r*_max_ is required, while all other steps in the determination of *R*_*T*_ remain analytical.

The formalism can be used for systems with a stable external forcing and a known periodicity. Examples are seasonality and the circadian rhythm which force the biological system by temperature and light conditions, and which have a regular period (i.e. of 365 days for seasons or 24 h for the circadian cycle).

Several methodologies were previously put forward to describe forced periodicities in biological systems. Analytical solutions for specific problems are proposed by Bacaer and Guernaoui (2006). However, in their approach for each new problem new solutions need to be constructed using the simplicity of the system. A general numerical approach using Floquet theory is put forward by Klausmeier (2008). This is not limited to specific problems. However, this technique does not provide an analytical framework for theoretical considerations. Several attempts for such analytical framework were discussed by Heesterbeek and Roberts (1995a, b). They investigated the suitability of the Floquet multiplier as a threshold quantity for epidemic growth. According to them, this quantity is probably the easiest threshold quantity to calculate numerically. They were not very satisfied with this quantity, because at that time an analytical Floquet framework was not available, in which analytical solutions and analytical approximations for this quantity could be derived.

We provided such Floquet framework in this paper. It is an analytical framework, including both analytical and numerical approximation methods. Within this framework the Floquet ratio is the natural threshold quantity. We offer a straightforward 5 step recipe, by which analytical solutions or analytical approximations of this threshold quantity could be derived. If numerical solutions are required, this is only needed in the fourth step for the calculation of the eigenvalue of the Floquet growth operator.

Within this natural framework we focused on the Earth’s rotation as the single driving force of the periodicity. In the future, this can be extended in two ways. Firstly, a forced periodicity from another cause could also be described within this framework. The phase variable $\varphi$ would have a different origin and therefor a different meaning, but the mathematical formalism remains the same. Secondly, we present an example containing the seasonal rhythm and the circadian rhythm and we dealt with that by ignoring the relative fast circadian rhythm. A combination of several forced periodicities could be treated within a multi mode Floquet approach (Leskes et al. 2010). For each periodicity a phase variable should be defined where after the framework presented in this paper provide the tools to describe such a system. Although the procedure to formulate the multi mode Floquet matrix is straightforward, it is laborious to operate due to the large number of terms.
